# Supplementary material for: Plasma sphingolipid abnormalities in neurodegenerative diseases
Source: PLoS One. 2022 Dec 16;17(12):e0279315. doi: 10.1371/journal.pone.0279315 (PMC9757566; doi:10.1371/journal.pone.0279315)
Supplement: S1 Table — Statistical methods: The metabolite level ratio of IPD, DLB, MSA, AD, or PSP to CNs. Statistical significance was examined using one-tailed Welch’s t tests (P < 0.05). Abbreviations: ceramide-1-phosphate (C1P), sphinganine-1-phosphate (SG1P), lysophosphatidic acid (LPA), lysophosphatidylcholine (LPC), lysophosphatidylethanolamine (LPE), lysophosphatidylglycerol (LPG), lysophosphatidylinositol (LPI), lysophosphatidylserine (LPS). (DOCX) [file pone.0279315.s001.docx]

**S1 Table. Plasma Other sphingolipids, Sphinganines, Gangliosides, Free fatty acids, Acylcarnitnes, Lysophospholipids Levels in Neurodegenerative Diseases.**

| cohort A |  |  | cohort B |  |  | cohort B |  |  |
| --- | --- | --- | --- | --- | --- | --- | --- | --- |
| PD vs CN |  |  | DLB vs CN |  |  | AD vs CN |  |  |
|  | ratio | p value |  | ratio | p value |  | ratio | p value |
| **other sphingolipids** |  |  | **other sphingolipids** |  |  | **other sphingolipids** |  |  |
| C1P | 1.1 | **0.0291** | C1P | 1.2 | **0.0004** | C1P | 1.3 | **0.0001** |
| sphingosine | 0.8 | **0.0436** | sphingosine | 0.7 | 0.0586 | sphingosine | 0.8 | 0.1119 |
| **sphinganines** |  |  | **sphinganines** |  |  | **sphinganines** |  |  |
| sphinganine | 0.8 | **0.0031** | sphinganine | 0.8 | **0.0191** | sphinganine | 0.8 | **0.0248** |
| SG1P | 0.9 | 0.0535 | SG1P | 0.8 | **0.0312** | SG1P | 0.8 | **0.0284** |
| **gangliosides** |  |  | **gangliosides** |  |  | **gangliosides** |  |  |
| GM3 | 1.1 | **0.0169** | GM3 | 1.2 | **0.0001** | GM3 | 1.3 | **<0.0001** |
| GD3 | 1.1 | **0.0483** | GD3 | 1.3 | **<0.0001** | GD3 | 1.4 | **<0.0001** |
| GD1 | 0.9 | 0.1471 | GD1 | 0.9 | 0.0927 | GD1 | 1.1 | 0.3198 |
| **free fatty acids** | 1.6 | **0.0174** | **free fatty acids** | 1.5 | 0.0502 | **free fatty acids** | 1.2 | 0.2940 |
| **acylcarnitnes** | 1.2 | 0.1048 | **acylcarnitnes** | 1.6 | **0.0014** | **acylcarnitnes** | 1.4 | 0.0580 |
| **lysophospholipids** |  |  | **lysophospholipids** |  |  | **lysophospholipids** |  |  |
| LPA | 0.9 | **0.0458** | LPA | 0.7 | **0.0092** | LPA | 0.9 | 0.3597 |
| LPC | 1 | 0.5986 | LPC | 0.8 | **0.0347** | LPC | 0.9 | 0.1295 |
| LPE | 1 | 0.5126 | LPE | 0.7 | **0.0034** | LPE | 0.9 | 0.2807 |
| LPG | 0.9 | **0.0032** | LPG | 0.8 | **0.0019** | LPG | 0.9 | 0.0453 |
| LPI | 0.9 | **0.0218** | LPI | 0.9 | 0.0749 | LPI | 1.1 | 0.3255 |
| LPS | 0.8 | **0.0366** | LPS | 0.6 | **0.0335** | LPS | 0.6 | 0.0734 |

| cohort C |  |  | cohort C |  |  | cohort C |  |  |
| --- | --- | --- | --- | --- | --- | --- | --- | --- |
| PD vs CN |  |  | PSP vs CN |  |  | MSA vs CN |  |  |
|  | ratio | p value |  | ratio | p value |  | ratio | p value |
| **other sphingolipids** |  |  | **other sphingolipids** |  |  | **other sphingolipids** |  |  |
| C1P | 1.3 | **0.0245** | C1P | 1.1 | 0.1851 | C1P | 1.2 | 0.1301 |
| sphingosine | 0.9 | 0.2337 | sphingosine | 0.7 | 0.0669 | sphingosine | 0.8 | 0.0902 |
| **sphinganines** |  |  | **sphinganines** |  |  | **sphinganines** |  |  |
| sphinganine | 0.9 | 0.8344 | sphinganine | 0.8 | 0.0718 | sphinganine | 0.9 | 0.1685 |
| SG1P | 0.7 | 0.0615 | SG1P | 0.7 | 0.0372 | SG1P | 0.7 | 0.0635 |
| **gangliosides** |  |  | **gangliosides** |  |  | **gangliosides** |  |  |
| GM3 | 1.3 | **<0.0001** | GM3 | 1.2 | **0.0005** | GM3 | 1.2 | **0.0012** |
| GD3 | 1.5 | **<0.0001** | GD3 | 1.2 | **0.0136** | GD3 | 1.1 | **<0.0001** |
| GD1 | 0.9 | 0.7283 | GD1 | 1.1 | 0.3171 | GD1 | 1.4 | 0.3579 |
| **free fatty acids** | 1.5 | 0.1049 | **free fatty acids** | 1.4 | 0.2088 | **free fatty acids** | 1.5 | 0.1559 |
| **acylcarnitnes** | 1.1 | 0.2606 | **acylcarnitnes** | 1.2 | 0.2046 | **acylcarnitnes** | 1.3 | 0.1451 |
| **lysophospholipids** |  |  | **lysophospholipids** |  |  | **lysophospholipids** |  |  |
| LPA | 1 | 0.5317 | LPA | 0.7 | **0.0308** | LPA | 1 | 0.5593 |
| LPC | 0.9 | **0.0335** | LPC | 0.9 | **0.0427** | LPC | 0.8 | **0.0128** |
| LPE | 0.8 | 0.0884 | LPE | 0.8 | 0.0532 | LPE | 0.7 | 0.0113 |
| LPG | 0.8 | 0.0645 | LPG | 0.8 | 0.0461 | LPG | 0.8 | **0.0423** |
| LPI | 1.1 | 0.1846 | LPI | 1 | 0.5590 | LPI | 1 | 0.5501 |
| LPS | 0.9 | 0.3106 | LPS | 0.7 | 0.1019 | LPS | 0.7 | 0.1078 |

Statistical methods: The metabolite level ratio of IPD, DLB, MSA, AD, or PSP to CNs. Statistical significance was examined using one-tailed Welch's t tests (P < 0.05).

Abbreviations: ceramide-1-phosphate (C1P), sphinganine-1-phosphate (SG1P), lysophosphatidic acid (LPA), lysophosphatidylcholine (LPC), lysophosphatidylethanolamine (LPE), lysophosphatidylglycerol (LPG), lysophosphatidylinositol (LPI), lysophosphatidylserine (LPS)
