# Supplementary material for: Plasma sphingolipid abnormalities in neurodegenerative diseases
Source: PLoS One. 2022 Dec 16;17(12):e0279315. doi: 10.1371/journal.pone.0279315 (PMC9757566; doi:10.1371/journal.pone.0279315)
Supplement: S2 Table — Statistical methods: The metabolite level ratio of IPD, DLB, MSA, AD, or PSP to CNs. Statistical significance was examined using one-tailed Welch’s t tests (P < 0.05). Abbreviations: platelet-activating factor (PAF). (DOCX) [file pone.0279315.s002.docx]

**S2 Table. Plasma Platelet-activating factor, Acylethanolamine, Thyroid hormone, Cholic acids, Steroids Levels in Neurodegenerative Diseases.**

| cohort A |  |  | cohort B |  |  | cohort B |  |  |
| --- | --- | --- | --- | --- | --- | --- | --- | --- |
| PD vs CN |  |  | DLB vs CN |  |  | AD vs CN |  |  |
|  | ratio | p value |  | ratio | p value |  | ratio | p value |
| **PAF** | 1 | 0.6096 | **PAF** | 0.9 | **0.0013** | **PAF** | 1 | 0.6249 |
| **acylethanolamine** | 1 | 0.5446 | **acylethanolamine** | 0.9 | 0.2251 | **acylethanolamine** | 0.9 | 0.2774 |
| **thyroid hormone** |  |  | **thyroid hormone** |  |  | **thyroid hormone** |  |  |
| thyroxine | 1.1 | **0.0222** | thyroxine | 1.1 | 0.0514 | thyroxine | 1.3 | **0.0009** |
| triiodothyronine | 1 | 0.6214 | triiodothyronine | 0.9 | 0.0681 | triiodothyronine | 1.2 | 0.1464 |
| **cholic acids** |  |  | **cholic acids** |  |  | **cholic acids** |  |  |
| ursodeoxycholic acid | 0.6 | 0.0826 | ursodeoxycholic acid | 1.4 | 0.2270 | ursodeoxycholic acid | 0.8 | 0.2251 |
| chenodeoxycholic acid | 0.8 | 0.6877 | chenodeoxycholic acid | 1.6 | 0.2008 | chenodeoxycholic acid | 1 | 0.5052 |
| cholic acid | 0.3 | 0.0508 | cholic acid | 1 | 0.5223 | cholic acid | 0.7 | 0.3086 |
| deoxycholic acid | 0.8 | 0.2792 | deoxycholic acid | 1.3 | 0.2218 | deoxycholic acid | 1 | 0.4672 |
| lithocholic acid | 0.7 | 0.0756 | lithocholic acid | 1.1 | 0.6199 | lithocholic acid | 0.6 | 0.1386 |
| **steroids** |  |  | **steroids** |  |  | **steroids** |  |  |
| corticosterone | 1.2 | 0.1974 | corticosterone | 1.2 | 0.1394 | corticosterone | 1.2 | 0.1417 |
| cortisol | 1.1 | 0.0854 | cortisol | 1.2 | 0.0521 | cortisol | 1.3 | **0.0051** |
| cortisone | 1.2 | **0.0054** | cortisone | 1 | 0.3228 | cortisone | 1 | 0.6957 |

| cohort C |  |  | cohort C |  |  | cohort C |  |  |
| --- | --- | --- | --- | --- | --- | --- | --- | --- |
| PD vs CN |  |  | PSP vs CN |  |  | MSA vs CN |  |  |
|  | ratio | p value |  | ratio | p value |  | ratio | p value |
| **PAF** | 0.9 | **0.0061** | **PAF** | 0.8 | 0.0036 | **PAF** | 0.8 | **0.0019** |
| **acylethanolamine** | 0.6 | 0.1959 | **acylethanolamine** | 0.6 | 0.1917 | **acylethanolamine** | 0.6 | 0.2118 |
| **thyroid hormone** |  |  | **thyroid hormone** |  |  | **thyroid hormone** |  |  |
| thyroxine | 1.3 | **0.0074** | thyroxine | 1.2 | **0.0135** | thyroxine | 1.2 | **0.0240** |
| triiodothyronine | 1.2 | 0.2475 | triiodothyronine | 1.1 | 0.4140 | triiodothyronine | 0.9 | 0.3577 |
| **cholic acids** |  |  | **cholic acids** |  |  | **cholic acids** |  |  |
| ursodeoxycholic acid | 0.4 | 0.0651 | ursodeoxycholic acid | 5.8 | 0.1491 | ursodeoxycholic acid | 0.4 | 0.0746 |
| chenodeoxycholic acid | 0.9 | 0.5749 | chenodeoxycholic acid | 1.6 | 0.2746 | chenodeoxycholic acid | 0.2 | 0.1051 |
| cholic acid | 0.6 | 0.3441 | cholic acid | 1.3 | 0.3896 | cholic acid | 0.2 | 0.2015 |
| deoxycholic acid | 0.8 | 0.6290 | deoxycholic acid | 1 | 0.5139 | deoxycholic acid | 0.5 | 0.1928 |
| lithocholic acid | 0.6 | 0.2232 | lithocholic acid | 0.5 | 0.1522 | lithocholic acid | 0.5 | 0.1422 |
| **steroids** |  |  | **steroids** |  |  | **steroids** |  |  |
| Corticosterone | 1 | 0.4586 | Corticosterone | 1.2 | 0.3038 | Corticosterone | 0.9 | 0.6421 |
| Cortisol | 1.2 | 0.1064 | Cortisol | 1.3 | **0.0143** | Cortisol | 1.2 | 0.1288 |
| Cortisone | 1.2 | **0.0295** | Cortisone | 1.2 | **0.0490** | Cortisone | 1.3 | **0.0295** |

Statistical methods: The metabolite level ratio of IPD, DLB, MSA, AD, or PSP to CNs. Statistical significance was examined using one-tailed Welch's t tests (P < 0.05).

Abbreviations: platelet-activating factor (PAF)
