# Supplementary material for: Plasma sphingolipid abnormalities in neurodegenerative diseases
Source: PLoS One. 2022 Dec 16;17(12):e0279315. doi: 10.1371/journal.pone.0279315 (PMC9757566; doi:10.1371/journal.pone.0279315)
Supplement: S3 Table — Pearson Correlation Coefficient was used to correlate between age and plasma S1P d16:1 levels, plasma S1P d18:1 levels, plasma MonCer d18:1 levels, or plasma LacCer d18:1 levels (P < 0.05). (DOCX) [file pone.0279315.s003.docx]

**S3 Table. Correlation Analysis between Age and Plasma S1P d16:1 levels, Plasma S1P d18:1 levels, Plasma MonCer d18:1 levels, or Plasma LacCer d18:1 levels in DLB and AD.**

| DLB |  | S1P d16:1 | S1P d18:1 | MonCer d18:1 | LacCer d18:1 |
| --- | --- | --- | --- | --- | --- |
|  | r | -0.12 | 0.091 | 0.343 | 0.197 |
|  | p value | 0.544 | 0.644 | 0.074 | 0.315 |
| AD |  | S1P d16:1 | S1P d18:1 | MonCer d18:1 | LacCer d18:1 |
|  | r | -0.203 | 0.093 | 0.237 | 0.219 |
|  | p value | 0.506 | 0.762 | 0.434 | 0.742 |

Pearson Correlation Coefficient was used to correlate between age and plasma S1P d16:1 levels, plasma S1P d18:1 levels, plasma MonCer d18:1 levels, or plasma LacCer d18:1 levels (P < 0.05).
