# Supplementary material for: Plasma sphingolipid abnormalities in neurodegenerative diseases
Source: PLoS One. 2022 Dec 16;17(12):e0279315. doi: 10.1371/journal.pone.0279315 (PMC9757566; doi:10.1371/journal.pone.0279315)
Supplement: Attachment — Submitted filename: Response to Reviewers20220918.docx [file pone.0279315.s004.docx]

Additional Editor Comments:

Major issue:

lines 190-213 (Results section on S1P): The authors repeat in words what is depicted in the figures. This is far too repetitive. What is the purpose of the figures, if all of the findings are given in words?

Example: " Plasma
S1P d16.1 levels were significantly lower in the IPD group of cohort A (plasma S1P d16.1 levels:
30 IPD vs. 28 CNs; 0.0091 (0.0103-0.00713) vs. 0.0115 (0.013-0.0103); t = 4.34, p < 0.0001) (Fig 1A),

Suggested way to rephrase the above sentence:
Plasma S1P d16.1 levels were significantly (p,0.0001) lower in the IPD group of cohort A (N=30) versus the control group (N=28) (Fig 1A)..."

Thank you for your suggestion. We agree with your opinion. We added sentences in Results section as you have indicated.

Plasma S1P d16.1 levels were significantly (p < 0.0001) lower in the IPD group of cohort A (N=30) versus the control group (N=28) (Fig 1A). Plasma S1P d16.1 levels were significantly (p < 0.0001) lower in the DLB group (N=28) versus the control group (N=15) (Fig 1B) and significantly (p < 0.0001) lower in the AD group (N=13) versus the control group (N=15) (Fig 1B). Plasma S1P d16.1 levels were significantly (p < 0.01) lower in the IPD group of cohort C (N=28) versus the control group (N=6) (Fig 1C), significantly (p < 0.01) lower in the MSA group (N=13) versus the control group (N=6) (Fig 1C), and significantly (p < 0.001) lower in the PSP group (N=16) versus the control group (N=6) (Fig 1C). Plasma S1P d18.1 levels were significantly (p < 0.05) lower in the IPD group of cohort A (N=30) versus the control group (N=28) (Fig 1D). Plasma S1P d18.1 levels were significantly (p < 0.01) lower in the DLB group (N=28) versus the control group (N=15) (Fig 1E) and significantly (p < 0.05) lower in the AD group (N=13) versus the control group (N=15) (Fig 1E). Plasma S1P d18.1 levels were significantly (p < 0.05) lower in the IPD group of cohort C (N=28) versus the control group (N=6) (Fig 1F), significantly (p < 0.05) lower in the MSA group (N=13) versus the control group (N=6) (Fig 1F), and significantly (p < 0.05) lower in the PSP group (N=16) versus the control group (N=6) (Fig 1F). (p13-14, lines 204-219)

Total plasma MonCer d18:1 levels were significantly (p < 0.01) higher in the IPD group of cohort A (N=30) versus the control group (N=28) (Fig 2A). Total plasma MonCer d18:1 levels were significantly (p < 0.01) higher in the DLB group (N=28) versus the control group (N=15) (Fig 2B) and significantly (p < 0.001) higher in the AD group (N=13) versus the control group (N=15) (Fig 2B). Total plasma MonCer d18:1 levels were significantly (p < 0.01) higher in the IPD group of cohort C (N=28) versus the control group (N=6) (Fig 2C), significantly (p < 0.05) higher in the MSA group (N=13) versus the control group (N=6) (Fig 2C), and significantly (p < 0.01) higher in the PSP group (N=16) versus the control group (N=6) (Fig 2C). (p15, lines 243-251)

Total plasma LacCer d18:1 levels were significantly (p < 0.01) higher in the IPD group of cohort A (N=30) versus the control group (N=28) (Fig 3A). Total plasma LacCer d18:1 levels were significantly (p < 0.001) higher in the DLB group (N=28) versus the control group (N=15) (Fig 3B) and significantly (p < 0.05) higher in the AD group (N=13) versus the control group (N=15) (Fig 3B). Total plasma LacCer d18:1 levels were significantly (p < 0.01) higher in the IPD group of cohort C (N=28) versus the control group (N=6) (Fig 3C), significantly (p < 0.01) higher in the MSA group (N=13) versus the control group (N=6) (Fig 3C), and significantly (p < 0.01) higher in the PSP group (N=16) versus the control group (N=6) (Fig 3C). (p16-17, lines 270-278)

Reviewers' comments:

Reviewer's Responses to Questions

Reviewer #1: This manuscript is interesting, but very preliminary. Only a few sphingolipids on the total species present in plasma were analyzed. Of course the analysis of plasma lipids from GBA1 associated neurodegenerative disease patients would be necessary, but I believe that more important would be the knowledge on the others, not considered sphingolipids.

1)
Information on the separation of GalCer from GlcCer is necessary to understand to which compound are referred the results.

Thank you for your important remarks.

Unfortunately, it has been reported that the majority of plasma Monohexylceramides (MonCer) is composed of plasma GlcCer [1], but it is difficult to completely separate plasma GalCer from plasma GlcCer with present methods. Thank you for your advice. As you have indicated, plasma MonCer is the sum of plasma GalCer and plasma GlcCer, so we described plasma GlcCer as plasma MonCer. In addition, Mielke et al. also described plasma GlcCer as GlcCer (a MonCer) in their PROS ONE manuscript [2]. Thus, we also described plasma GlcCer as GlcCer (a MonCer) in reference to their PROS ONE manuscript.

In response to your suggestion, we have added text to the Abstract, Results and Discussion sections, and have referred to plasma GlcCer as plasma MonCer or GlcCer (a MonCer) in this manuscript.

(2) Plasma monohexylceramide (MonCer) and lactosylceramide (LacCer) were significantly higher in all neurodegenerative disease groups (IPD, DLB, MSA, AD, and PSP) than in the CN group. (3) Plasma MonCer levels were significantly positively correlated with plasma LacCer levels in all enrolled groups. **Conclusion**: S1P, Glucosylceramide (GlcCer), the main component of MonCer, and LacCer are sphingolipids that are biosynthesized from ceramide. (p3, lines 48-52)

Plasma Monohexylceramide (MonCer) levels in neurodegenerative diseases

Total plasma MonCer d18:1 levels were compared between the CN group and the IPD, DLB, MSA, AD, and PSP groups. Total plasma MonCer d18:1 levels were measured by summing levels of 13 types of MonCer d18:1: MonCer (d18:1/14:0), MonCer (d18:1/16:0), MonCer (d18:1/16:1), MonCer (d18:1/18:0), MonCer (d18:1/18:1), MonCer (d18:1/20:0), MonCer (d18:1/20:1), MonCer (d18:1/22:0), MonCer (d18:1/22:1), MonCer (d18:1/22:2), MonCer (d18:1/24:0), MonCer (d18:1/24:1) and MonCer (d18:1/24:2). Statistical significance was examined using one-tailed Welch's t tests. Total plasma MonCer d18:1 levels were significantly (p < 0.01) higher in the IPD group of cohort A (N=30) versus the control group (N=28) (Fig 2A). Total plasma MonCer d18:1 levels were significantly (p < 0.01) higher in the DLB group (N=28) versus the control group (N=15) (Fig 2B) and significantly (p < 0.001) higher in the AD group (N=13) versus the control group (N=15) (Fig 2B). Total plasma MonCer d18:1 levels were significantly (p < 0.01) higher in the IPD group of cohort C (N=28) versus the control group (N=6) (Fig 2C), significantly (p < 0.05) higher in the MSA group (N=13) versus the control group (N=6) (Fig 2C), and significantly (p < 0.01) higher in the PSP group (N=16) versus the control group (N=6) (Fig 2C). These results indicated that plasma MonCer levels were significantly higher in all neurodegenerative disease groups (IPD, DLB, MSA, AD, and PSP) than in the CN group.

**Fig 2. Plasma MonCer Levels in Neurodegenerative Diseases.** (A) Plasma MonCer d18:1 levels were significantly higher in the IPD group of cohort A (p < 0.01) than in the CN group. (B) Plasma MonCer d18:1 levels were significantly higher in the DLB group (p < 0.01) and AD group (p < 0.001) than in the CN group. (C) Plasma MonCer d18:1 levels were significantly higher in the IPD group of cohort C (p < 0.01), MSA group (p < 0.05) and PSP group (p < 0.01) than in the CN group. (p15-16, lines 235-260)

**Correlation between total plasma MonCer levels and total plasma LacCer levels.**

Pearson Correlation Coefficient was used to correlate total plasma MonCer d18:1 levels and total plasma LacCer d18:1 levels in all enrolled groups. Total plasma MonCer d18:1 levels were significantly positively correlated with total plasma LacCer d18:1 levels (r = 0.5802, p < 0.0001) (Fig 4) in all enrolled groups. These results suggest that an increase in plasma MonCer may be directly related to an increase in LacCer in all enrolled groups.

**Fig 4. Correlation between total plasma MonCer levels and total plasma LacCer levels.** (A) Total plasma MonCer d18:1 levels were significantly positively correlated with total plasma LacCer d18:1 levels (r = 0.5802, p < 0.0001) in all enrolled groups.

(p17-18, lines 289-298)

In this study, we found that plasma S1P levels were significantly lower and plasma MonCer and LacCer levels were significantly higher in the neurodegenerative disease groups than in the CN group in a plasma lipidomics study. Glucosylceramide (GlcCer) and galactosylceramide (GalCer) are isomers, and MonCer is the sum of both compounds. Although it is difficult to completely separate plasma GalCer and plasma GlcCer from plasma MonCer in present method, it has been shown that the majority of plasma MonCer is composed of plasma GlcCer [1]. (p19, lines 312-317)

Plasma GlcCer (a MonCer) levels have been shown to be significantly elevated in PD, autopsy-confirmed DLB, and autopsy-confirmed AD groups [2,3]. However, there has been no comprehensive analysis of plasma GlcCer (a MonCer) levels in neurodegenerative diseases such as synucleinopathies and tauopathies. Therefore, we analyzed plasma GlcCer (a MonCer) in those with neurodegenerative diseases using lipidomics in this study. We found that the plasma GlcCer (a MonCer) levels were significantly higher in all neurodegenerative disease groups (IPD, DLB, MSA, AD, and PSP) than in the CN group. The elevated plasma GlcCer (a MonCer) levels in individuals with IPD, probable DLB, and probable AD in this study were in good accordance with the results of previous studies [2,3]. There have been no reports of abnormal plasma GlcCer (a MonCer) levels in MSA and PSP. In this study, we found elevated plasma GlcCer (a MonCer) levels in individuals not only with LB diseases or AD but also with MSA or PSP, suggesting that abnormalities in plasma GlcCer (a MonCer) metabolism are also commonly observed in synucleinopathies, amyloidopathies and tauopathies. (p21, lines 359-371)

2)
Quantitative data are necessary to understand the yield of extraction and the correct determination and comparison within samples.

We agree with your opinion. Thank you for your important remarks.

In present lipidomic analysis, the coefficient of variation (CV) ranged from 4.4 to 9.7%, with a mean of 6.7%. Because of the variability of metabolites values, present method should be compared with healthy subjects for each cohort. Therefore, the relative areas were used as metabolites values in this study.

Based on what you have pointed out, we added sentences in Discussion section.

Seventh, Quantitative data were not used as metabolites values in this study. In present lipidomic analysis, the coefficient of variation (CV) ranged from 4.4 to 9.7%, with a mean of 6.7%. Because of the variability of metabolites values, present method should be compared with healthy subjects for each cohort [4,5]. Therefore, the relative areas were used as metabolites values in this study. (p24, lines 427-431)

Reviewer #2: General Comment to authors
The authors examine plasma sphingolipid in several neurodegenerative diseases and show striking changes in sphingosine-1-phosphate and GluCer and LacCer species. However, the small sample sizes and the significant age differences in cohort B are concerns, given that age is a significant risk factor in Alzheimer’s disease.

Specific Comments to Authors

1)

Interestingly, sphingolipids change in all neurodegenerative diseases examined. How do the changes in S1P, GluCer, and LacCer relate to known changes in fluid biomarkers such as plasma amyloid beta 42 and amyloid beta 40 ratio, and plasma ptau levels?. It would be necessary also to measure these fluid biomarkers and not merely rely on MMSE, given the small sample sizes.

Thank you for your suggestion and your important remarks. Since our laboratory could analyze plasma p-tau levels, we analyzed plasma p-tau levels. Pearson Correlation Coefficient was used to correlate plasma p-tau levels and plasma S1P d16.1 levels, plasma S1P d18.1 levels, total plasma MonCer d18:1 levels or total plasma LacCer d18:1 levels in all enrolled groups. Correlation between plasma p-tau levels and plasma S1P d16.1 levels (p = 0.509), plasma S1P d18.1 levels (p = 0.468), plasma MonCer d18:1 levels (p = 0.767), or plasma LacCer d18:1 levels (p = 0.999) showed no correlation.

Based on your suggestion, we added sentences in Results section, Materials and Methods section.

**Correlation between plasma p-tau levels and plasma S1P levels, total plasma MonCer levels or total plasma LacCer levels.**

To investigate the association between AD-associated protein and sphingolipids, Pearson Correlation Coefficient was used to correlate plasma p-tau levels and plasma S1P d16.1 levels, plasma S1P d18.1 levels, total plasma MonCer d18:1 levels or total plasma LacCer d18:1 levels in all enrolled groups. Correlation between plasma p-tau levels and plasma S1P d16.1 levels (p = 0.509), plasma S1P d18.1 levels (p = 0.468), plasma MonCer d18:1 levels (p = 0.767), or plasma LacCer d18:1 levels (p = 0.999) showed no correlation. (p18, lines 300-307)

**Simoa™ Assay**

Plasma samples stored at -80°C were thawed and centrifuged at 10,000 x g for 5 minutes. Samples were diluted in advance with the Sample Diluent provided with Assay Kit and applied to the plate. The assay was performed one sample at a time. Simoa™ p-Tau181 Advantage Kit (Quanterix, #103377, Billerica, MA, USA) were used to measure plasma p-Tau181. Measurements were performed according to the instructions for kit. (p12, lines 185-190)

*It was proposed by reviewer 1 to show information on the separation of GalCer from GlcCer. Glucosylceramide (GlcCer) and galactosylceramides (GalCer) are isomers, and MonCer is the sum of both compounds. Although it is difficult to completely separate plasma GalCer and plasma GlcCer from plasma MonCer in present method, it has been shown that the majority of plasma MonCer is composed of plasma GlcCer. In addition, Mielke et al. also described plasma GlcCer as GlcCer (a MonCer) in their PROS ONE manuscript [2]. Thus, we also described plasma GlcCer as GlcCer (a MonCer) in reference to their PROS ONE manuscript.

2)

How do the plasma changes in sphingolipids relate to brain and CSF changes?

Thank you for your suggestion. We added sentences in Discussion section as you have indicated.

In previous reports, the increase and decrease in CSF sphingolipids and blood sphingolipids have coincided, suggesting that blood sphingolipids may reflect the dynamics of the central nervous system in neurodegenerative diseases. (p19, lines 322-324)

3)

The age of the CN population (cohort B) is significantly lower than that of the DLB and AD populations. Since age is a significant risk factor for AD, it is unclear whether the changes are AD-related or age-related. Additional analyses with age as a variable may help tease out whether these data are age-related or pathological.

Thank you for your important remarks. Based on your suggestion, we have performed additional statistical analysis.

Pearson Correlation Coefficient was used to correlate age and plasma S1P d16:1 levels, plasma S1P d18:1 levels, plasma MonCer d18:1 levels, or plasma LacCer d18:1 levels in DLB or AD. In DLB, correlation between age and plasma S1P d16:1 levels (p = 0.544), plasma S1P d18:1 levels (p = 0.644), plasma MonCer d18:1 levels (p = 0.074), or plasma LacCer d18:1 levels (p = 0.315) showed no correlation. In AD, correlation between age and plasma S1P d16:1 levels (p = 0.506), plasma S1P d18:1 levels (p = 0.762), plasma MonCer d18:1 levels (p = 0.434), or plasma LacCer d18:1 levels (p = 0.742) showed no correlation. Therefore, it was inferred that changes in plasma S1P d16:1 levels, plasma S1P d18:1 levels, plasma MonCer d18:1 levels or plasma LacCer d18:1 levels were inferred to be disease-induced changes in AD or DLB.

We added sentences in Discussion section as you have indicated.

Fifth, cohort B was not an age-matched study. In DLB and AD, correlation analysis between age and plasma S1P d16:1 levels, plasma S1P d18:1 levels, plasma MonCer d18:1 levels, or plasma LacCer d18:1 levels showed no correlation (data not shown). Thus, changes in plasma S1P d16:1 levels, plasma S1P d18:1 levels, plasma MonCer d18:1 levels or plasma LacCer d18:1 levels were inferred to be disease-induced changes in AD or DLB. (p24, lines 421-425)

4)

What is the recovery of sphingolipids extracted with the SPE column (page 11)? What internal standards were used, and how quantitative were these standards?

Thank you for your suggestion. Based on your suggestion, we added sentences in Materials and Methods section.

The average recovery of sphingolipids extracted with the SPE column is 88% (range 68% to 99.9%). (p11, lines 165-166)

Internal standards are used at a concentration of 50 uM. Cer/Sph Mixture II (Avanti Polar Lipids, LM6005, Birmingham, AL, USA) was used as internal standards of sphingolipid. (p12, lines 181-183)

5)

Is there a correlation/link between the decrease in S1P and the increase in GlcCer and LacCer in plasma?

Thank you for your suggestion. We agree with your opinion. Based on your suggestion, we have performed additional statistical analysis.

Pearson Correlation Coefficient was used to correlate plasma MonCer levels and plasma LacCer levels and plasma S1P levels in all enrolled groups. Total plasma MonCer d18:1 levels were significantly positively correlated with total plasma LacCer d18:1 levels (r = 0.5802, p < 0.0001). There was no difference between total plasma LacCer d18:1 levels and plasma S1P d16:1 levels (r = -0.0092, p = 0.9091) or plasma S1P d18:1 levels (r = 0.0989, p = 0.2195). There was no difference between total plasma MonCer d18:1 levels and plasma S1P d16:1 levels (r = 0.0719, p = 0.3721), but total plasma MonCer d18:1 levels were significantly slight positively correlated with plasma S1P d18:1 levels (r = 0.2480, p = 0.0028). These results suggest that an increase in plasma MonCer d18:1 levels may be directly related to an increase in LacCer d18:1 levels in all enrolled groups.

We found interesting results, as you pointed out. Thank you very much.

We added sentences in Discussion section, based on the above results.

**Correlation between total plasma MonCer levels and total plasma LacCer levels.**

Pearson Correlation Coefficient was used to correlate total plasma MonCer d18:1 levels and total plasma LacCer d18:1 levels in all enrolled groups. Total plasma MonCer d18:1 levels were significantly positively correlated with total plasma LacCer d18:1 levels (r = 0.5802, p < 0.0001) (Fig 4) in all enrolled groups. These results suggest that an increase in plasma MonCer may be directly related to an increase in LacCer in all enrolled groups.

**Fig 4. Correlation between total plasma MonCer levels and total plasma LacCer levels.** (A) Total plasma MonCer d18:1 levels were significantly positively correlated with total plasma LacCer d18:1 levels (r = 0.5802, p < 0.0001) in all enrolled groups. (p17-18, lines 289-298)

What enzyme pathways may cause the decrease in S1P and the increase in GluCer and LacCer?

Thank you for your suggestion. Based on your suggestion, we added sentences in Discussion section.

GCS is GlcCer synthase, BGTase6 is LacCer synthase, and SPHK is S1P synthase. These indicate that increased GlcCer and LacCer are caused by increased function of GCS and BGTase6, respectively, and decreased S1P is caused by a relative loss of function of SPHK. (p19, lines 319-321)

6)

The limitations (page 22) should include that this is a small discovery/cross sectional study that can not account for multiple comparisons for several analytes detected in plasma.

Thank you for your suggestion. We added sentences in Discussion section as you have indicated.

Sixth, this study is a small discovery/cross sectional study that could not account for multiple comparisons for several analytes detected in plasma. (p24, lines 425-427)

1.	Xu H, Boucher FR, Nguyen TT, Taylor GP, Tomlinson JJ, Ortega RA, et al. DMS as an orthogonal separation to LC/ESI/MS/MS for quantifying isomeric cerebrosides in plasma and cerebrospinal fluid. J Lipid Res. 2019;60: 200-211.

2.	Mielke MM, Maetzler W, Haughey NJ, Bandaru VV, Savica R, Deuschle C, et al. Plasma ceramide and glucosylceramide metabolism is altered in sporadic Parkinson's disease and associated with cognitive impairment: a pilot study. PLoS One. 2013;8: e73094.

3.	Savica R, Murray ME, Persson XM, Kantarci K, Parisi JE, Dickson DW, et al. Plasma sphingolipid changes with autopsy-confirmed Lewy Body or Alzheimer's pathology. Alzheimers Dement (Amst). 2016;3: 43-50.

4.	Mori A, Ishikawa KI, Saiki S, Hatano T, Oji Y, Okuzumi A, et al. Plasma metabolite biomarkers for multiple system atrophy and progressive supranuclear palsy. PLoS One. 2019;14: e0223113.

5.	Saiki S, Sasazawa Y, Fujimaki M, Kamagata K, Kaga N, Taka H, et al. A metabolic profile of polyamines in parkinson disease: A promising biomarker. Ann Neurol. 2019;86: 251-263.
