# Supplementary material for: Plasma sphingolipid abnormalities in neurodegenerative diseases
Source: PLoS One. 2022 Dec 16;17(12):e0279315. doi: 10.1371/journal.pone.0279315 (PMC9757566; doi:10.1371/journal.pone.0279315)
Supplement: Attachment — Submitted filename: Response to reviewers20221113.docx [file pone.0279315.s005.docx]

*Thank you for submitting your manuscript to PLOS ONE. After careful consideration, we feel that it has merit but does not fully meet PLOS ONE’s publication criteria as it currently stands. Therefore, we invite you to submit a revised version of the manuscript that addresses the points raised during the review process.

1) Is the manuscript technically sound, and do the data support the conclusions?*

*In response to this question, reviewer #3 says "partly" while reviewer #1 had no response.*

Thanks for the suggestion. We have made the new revised manuscript to improve on original manuscript.

*2) Have the authors made all data underlying the findings in their manuscript fully available?*

*Reviewer #3 says "no."*

Thank you for your suggestion. Supporting information (S1 Table, S2 Table, and S3 Table) and additional Table (Table 2 and Table 3) has been added to the new revised manuscript to improve on original manuscript.

*3) Reviewer #1 says that his comments pertaining to the original manuscript were not addressed:*

*(i) I believe that more important would be the knowledge on the others, not considered sphingolipids.*

*Editor: Point 3(i) has not been addressed, and this was also brought up by reviewer #3.*

Thank you for your suggestion. We have described a comprehensive knowledge of lipid abnormalities reported in neurodegenerative diseases.

We added sentences in Discussion section. Changes in the revised manuscript are highlighted in yellow.

Recessive mutations in the GBA1 (glucocerebrosidase) gene cause Gaucher disease. Heterozygous GBA1 mutation carriers exhibit much greater incidence of PD than the general population [1,2]. Likewise, mutations in the NPC1 (NPC intracellular cholesterol transporter 1) and SMPD1 (sphingomyelin phosphodiesterase 1) genes, which cause Niemann-Pick disease, have been shown to be risk genes for IPD [3,4]. One of the phospholipase A2 members, PLA2G6 or iPLA2-VIA/iPLA2β, has been isolated as the gene responsible for an autosomal recessive form of PD linked to the PARK14 locus [5]. Compared to the most common e3 isoform, the e4 isoform of ApoE (ApoE4) is the strongest genetic risk factor for late-onset AD [6]. β amyloid accumulation in NPC1 (NPC intracellular cholesterol transporter 1) gene, which cause Niemann-Pick type C, mutant cells and NPC mouse brain suggests the association between cholesterol metabolism and AD [7]. As described, several lipid-related genes have been reported as risk genes or causative genes in AD and IPD. In addition, various lipid abnormalities have been reported in IPD and AD, such as fatty acids, glycerolipids, glycerophospholipids, sphingolipids, sterols, and lipoproteins [8,9]. However, it is still unclear which lipid metabolism abnormalities play the most important role in neurodegenerative diseases. Plasma lipidomics is an unbiased method and can find important lipids in neurodegenerative diseases. For this reason, plasma lipidomics was performed in neurodegenerative diseases in this study. (p25, lines 363-379)

*(ii) Information on the separation of GalCer from GlcCer is necessary to understand to which compound are referred the results.*

*Editor: I accept your explanation on the difficulties in separating GalCer from GlcCer.*

Thank you for your acceptance.

*(iii) Quantitative data are necessary to understand the yield of extraction and the correct determination and comparison within samples.*

*Editor: The reviewer was not satisfied as to your response to 3(iii).*

Thank you for your suggestion. We measured relative areas as quantitative value based on PROS ONE manuscript [10]. Lipidomics has the variability of metabolite values in each study. For this reason, each metabolite should be normalized based on the internal standard (IS) level and sample volume. The normalized each metabolite was represented as relative area and used as the quantitative value. Additionally, information on IS was described in Materials and Methods section.

We changed sentence in Materials and Methods, and Discussion section. Changes in the revised manuscript are highlighted in yellow.

Target metabolites are divided into categories (fatty acids, acylcarnitines, oxylipins, lysophospholipids, platelet-activating factors, glycosphingolipids, sphinganines, sphingosines, and steroids) according to their physical properties, and the recovery rate is corrected using the corresponding IS (Internal standards). Based on these reports, these IS were selected [11-13]. The recovery rate of analytes during extraction ranged from 68% to 129%, with a mean of 96%. IS coefficient of variation ranged from 4.4 to 9.7%, with a mean of 6.7%. The peak area of each metabolite was then normalized based on IS level and sample volume for relative quantification. The normalized each metabolite was represented as relative area and used as the quantitative value based on previous reports [10,14]. (p12, lines 182-191)

Seventh, relative area was used in this study as the quantitative value for each metabolite based on previous reports [10,14]. Lipidomics has the variability of metabolite values in each study. For this reason, each metabolite should be normalized based on the IS level and sample volume. The normalized each metabolite was represented as relative area and used as the quantitative value. (p32-33, lines 518-522)

*4)  Reviewer#3 comments:*

*(i)The authors do not provide an explanation of which enzyme pathways can be affected in the observed alterations. It would provide important insights if the authors can perform additional experiments in testing the protein levels of the enzymes involved in the pathways.*

*Editor: Please respond to 4(i). Testing protein levels of the enzymes could be helpful. Can this be done?*

Thank you for your suggestion. However, in this study we cannot perform additional experiments in testing the protein levels of the enzymes involved in sphingolipid pathways.

We added sentences in Discussion section. Changes in the revised manuscript are highlighted in yellow.

Sixth, in this study the protein levels of the enzymes involved in sphingolipid pathways were not evaluated in all enrolled patients. The protein levels of the enzymes involved in sphingolipid pathways need to be performed in future studies. (p32, lines 516-518)

*(ii) What is the outcome of other lipids that were analyzed in this lipidomics analyses? For completeness, they should also include those, even if no differences were observed. If no other lipids were analyzed, the authors should include a reasoning why they only tested these lipids.*

*Editor:* *You should include the results for other lipids tested.*

Thank you for your suggestion. We included the results for other lipids tested.

We added sentences in Results, Discussion, Supporting information section. We added S1 Table and S2 Table. Changes in the revised manuscript are highlighted in yellow.

Plasma other lipid metabolite levels in neurodegenerative diseases.

Plasma other lipid metabolite (other sphingolipids, sphinganines, gangliosides, free fatty acids, acylcarnitnes, lysophospholipids, platelet-activating factor, acylethanolamine, thyroid hormone, cholic acids, and steroids) levels were compared between the CN group and the IPD, DLB, MSA, AD and PSP groups. Oxylipins were not statistically analyzed because it is considered unsuitable for statistical analysis due to the large number of undetectable samples. Statistical significance was examined using one-tailed Welch’s t tests. Plasma ceramide-1-phosphate (C1P) levels were significantly higher in the PD, DLB, and AD groups versus the control group (S1 table). Plasma GM3 ganglioside and GD3 ganglioside levels were significantly higher in all neurodegenerative disease groups (IPD, DLB, MSA, AD, and PSP) versus the control group (S1 table). Plasma lysophosphatidic acid, lysophosphatidylcholine, lysophosphatidylethanolamine, lysophosphatidylglycerol, lysophosphatidylserine levels were lower in DLB group versus the control group (S1 table). Plasma cortisone levels were significantly higher in the PD, MSA and PSP groups versus the control group (S2 Table). (p19-20, lines 332-345)

In this study, we found that plasma GM3 and GD3 ganglioside levels were significantly higher in the neurodegenerative disease groups than in the CN group. Gangliosides are lipids classified as sphingolipids. GM3 ganglioside is the starting material for gangliosides, which are biosynthesized by the binding of sialic acid to LacCer [15,16]. Previously, plasma GM3 ganglioside levels have been shown to be elevated in PD [17]. The elevated plasma GM3 ganglioside levels in individuals with IPD in this study were in good accordance with the results of previous study. GD3 ganglioside is the gangliosides, which are biosynthesized by the binding of sialic acid to GM3 ganglioside [15,16]. GM3 and GD3 gangliosides are components of lipid rafts and are implicated in cell death [18,19]. Abnormalities in lipid rafts are also considered to be one of the major causes of neurodegenerative diseases [20]. Homozygous knockout mice for B4galnt1, a ganglioside synthase, have been shown to exhibit PD-like motor deficits and cause dopaminergic neuron degeneration [21]. Taken together, these results suggest that elevated plasma GM3 and GD3 gangliosides may reflect abnormal lipid rafts in neurodegenerative diseases. In this study, we found that plasma C1P levels were significantly higher in the IPD, DLB, and AD groups than in the CN group. C1P is classified as a sphingolipid, a lipid mainly involved in cell survival and inflammation [22,23]. Neuroinflammation is also considered to be a one of the major causes in PD, DLB and AD [24-26]. Therefore, elevated C1P may reflect neuroinflammation in these diseases. (p30-31, lines 472-489)

**S1 Table. Plasma other sphinogolipids, sphinganines, gangliosides, free fatty acids, acylcarnitnes, lysophospholipids levels in neurodegenerative diseases.**

Statistical methods: The metabolite level ratio of IPD, DLB, MSA, AD, or PSP to CNs. Statistical significance was examined using one-tailed Welch's t tests (P < 0.05).

Abbreviations: ceramide-1-phosphate (C1P), sphinganine-1-phosphate (SG1P), lysophosphatidic acid (LPA), lysophosphatidylcholine (LPC), lysophosphatidylethanolamine (LPE), lysophosphatidylglycerol (LPG), lysophosphatidylinositol (LPI), lysophosphatidylserine (LPS) (p46, lines 758-764)

**S2 Table. Plasma platelet-activating factor, acylethanolamine, thyroid hormone, cholic acids, steroids levels in neurodegenerative diseases.**

Statistical methods: The metabolite level ratio of IPD, DLB, MSA, AD, or PSP to CNs. Statistical significance was examined using one-tailed Welch's t tests (P < 0.05).

Abbreviations: platelet-activating factor (PAF) (p46, lines 765-769)

**S1 Table. Plasma Other sphinogolipids, Sphinganines, Gangliosides, Free fatty acids, Acylcarnitnes, Lysophospholipids Levels in Neurodegenerative Diseases.**

| cohort A |  |  | cohort B |  |  | cohort B |  |  |
| --- | --- | --- | --- | --- | --- | --- | --- | --- |
| PD vs CN |  |  | DLB vs CN |  |  | AD vs CN |  |  |
|  | ratio | p value |  | ratio | p value |  | ratio | p value |
| **other sphinogolipids** |  |  | **other sphinogolipids** |  |  | **other sphinogolipids** |  |  |
| C1P | 1.1 | **0.0291** | total C1P | 1.2 | **0.0004** | total C1P | 1.3 | **0.0001** |
| sphingosine | 0.8 | **0.0436** | sphingosine | 0.7 | 0.0586 | sphingosine | 0.8 | 0.1119 |
| **sphinganines** |  |  | **sphinganines** |  |  | **sphinganines** |  |  |
| sphinganine | 0.8 | **0.0031** | sphinganine | 0.8 | **0.0191** | sphinganine | 0.8 | **0.0248** |
| SG1P | 0.9 | 0.0535 | SG1P | 0.8 | **0.0312** | SG1P | 0.8 | **0.0284** |
| **gangliosides** |  |  | **gangliosides** |  |  | **gangliosides** |  |  |
| GM3 | 1.1 | **0.0169** | GM3 | 1.2 | **0.0001** | GM3 | 1.3 | **<0.0001** |
| GD3 | 1.1 | **0.0483** | GD3 | 1.3 | **<0.0001** | GD3 | 1.4 | **<0.0001** |
| GD1 | 0.9 | 0.1471 | GD1 | 0.9 | 0.0927 | GD1 | 1.1 | 0.3198 |
| **free fatty acids** | 1.6 | **0.0174** | **free fatty acids** | 1.5 | 0.0502 | **free fatty acids** | 1.2 | 0.2940 |
| **acylcarnitnes** | 1.2 | 0.1048 | **acylcarnitnes** | 1.6 | **0.0014** | **acylcarnitnes** | 1.4 | 0.0580 |
| **lysophospholipids** |  |  | **lysophospholipids** |  |  | **lysophospholipids** |  |  |
| LPA | 0.9 | **0.0458** | LPA | 0.7 | **0.0092** | LPA | 0.9 | 0.3597 |
| LPC | 1 | 0.5986 | LPC | 0.8 | **0.0347** | LPC | 0.9 | 0.1295 |
| LPE | 1 | 0.5126 | LPE | 0.7 | **0.0034** | LPE | 0.9 | 0.2807 |
| LPG | 0.9 | **0.0032** | LPG | 0.8 | **0.0019** | LPG | 0.9 | 0.0453 |
| LPI | 0.9 | **0.0218** | LPI | 0.9 | 0.0749 | LPI | 1.1 | 0.3255 |
| LPS | 0.8 | **0.0366** | LPS | 0.6 | **0.0335** | LPS | 0.6 | 0.0734 |

| cohort C |  |  | cohort C |  |  | cohort C |  |  |
| --- | --- | --- | --- | --- | --- | --- | --- | --- |
| PD vs CN |  |  | PSP vs CN |  |  | MSA vs CN |  |  |
|  | ratio | p value |  | ratio | p value |  | ratio | p value |
| **other sphinogolipids** |  |  | **other sphinogolipids** |  |  | **other sphinogolipids** |  |  |
| C1P | 1.3 | **0.0245** | C1P | 1.1 | 0.1851 | C1P | 1.2 | 0.1301 |
| sphingosine | 0.9 | 0.2337 | sphingosine | 0.7 | 0.0669 | sphingosine | 0.8 | 0.0902 |
| **sphinganines** |  |  | **sphinganines** |  |  | **sphinganines** |  |  |
| sphinganine | 0.9 | 0.8344 | sphinganine | 0.8 | 0.0718 | sphinganine | 0.9 | 0.1685 |
| SG1P | 0.7 | 0.0615 | SG1P | 0.7 | 0.0372 | SG1P | 0.7 | 0.0635 |
| **gangliosides** |  |  | **gangliosides** |  |  | **gangliosides** |  |  |
| GM3 | 1.3 | **<0.0001** | GM3 | 1.2 | **0.0005** | GM3 | 1.2 | **0.0012** |
| GD3 | 1.5 | **<0.0001** | GD3 | 1.2 | **0.0136** | GD3 | 1.1 | **<0.0001** |
| GD1 | 0.9 | 0.7283 | GD1 | 1.1 | 0.3171 | GD1 | 1.4 | 0.3579 |
| **free fatty acids** | 1.5 | 0.1049 | **free fatty acids** | 1.4 | 0.2088 | **free fatty acids** | 1.5 | 0.1559 |
| **acylcarnitnes** | 1.1 | 0.2606 | **acylcarnitnes** | 1.2 | 0.2046 | **acylcarnitnes** | 1.3 | 0.1451 |
| **lysophospholipids** |  |  | **lysophospholipids** |  |  | **lysophospholipids** |  |  |
| LPA | 1 | 0.5317 | LPA | 0.7 | **0.0308** | LPA | 1 | 0.5593 |
| LPC | 0.9 | **0.0335** | LPC | 0.9 | **0.0427** | LPC | 0.8 | **0.0128** |
| LPE | 0.8 | 0.0884 | LPE | 0.8 | 0.0532 | LPE | 0.7 | 0.0113 |
| LPG | 0.8 | 0.0645 | LPG | 0.8 | 0.0461 | LPG | 0.8 | **0.0423** |
| LPI | 1.1 | 0.1846 | LPI | 1 | 0.5590 | LPI | 1 | 0.5501 |
| LPS | 0.9 | 0.3106 | LPS | 0.7 | 0.1019 | LPS | 0.7 | 0.1078 |

Statistical methods: The metabolite level ratio of IPD, DLB, MSA, AD, or PSP to CNs. Statistical significance was examined using one-tailed Welch's t tests (P < 0.05).

Abbreviations: ceramide-1-phosphate (C1P), sphinganine-1-phosphate (SG1P), lysophosphatidic acid (LPA), lysophosphatidylcholine (LPC), lysophosphatidylethanolamine (LPE), lysophosphatidylglycerol (LPG), lysophosphatidylinositol (LPI), lysophosphatidylserine (LPS)

**S2 Table. Plasma Platelet-activating factor, Acylethanolamine, Thyroid hormone, Cholic acids, Steroids Levels in Neurodegenerative Diseases.**

| cohort A |  |  | cohort B |  |  | cohort B |  |  |
| --- | --- | --- | --- | --- | --- | --- | --- | --- |
| PD vs CN |  |  | DLB vs CN |  |  | AD vs CN |  |  |
|  | ratio | p value |  | ratio | p value |  | ratio | p value |
| **PAF** | 1 | 0.6096 | **PAF** | 0.9 | **0.0013** | **PAF** | 1 | 0.6249 |
| **acylethanolamine** | 1 | 0.5446 | **acylethanolamine** | 0.9 | 0.2251 | **acylethanolamine** | 0.9 | 0.2774 |
| **thyroid hormone** |  |  | **thyroid hormone** |  |  | **thyroid hormone** |  |  |
| thyroxine | 1.1 | **0.0222** | thyroxine | 1.1 | 0.0514 | thyroxine | 1.3 | **0.0009** |
| triiodothyronine | 1 | 0.6214 | triiodothyronine | 0.9 | 0.0681 | triiodothyronine | 1.2 | 0.1464 |
| **cholic acids** |  |  | **cholic acids** |  |  | **cholic acids** |  |  |
| ursodeoxycholic acid | 0.6 | 0.0826 | ursodeoxycholic acid | 1.4 | 0.2270 | ursodeoxycholic acid | 0.8 | 0.2251 |
| chenodeoxycholic acid | 0.8 | 0.6877 | chenodeoxycholic acid | 1.6 | 0.2008 | chenodeoxycholic acid | 1 | 0.5052 |
| cholic acid | 0.3 | 0.0508 | cholic acid | 1 | 0.5223 | cholic acid | 0.7 | 0.3086 |
| deoxycholic acid | 0.8 | 0.2792 | deoxycholic acid | 1.3 | 0.2218 | deoxycholic acid | 1 | 0.4672 |
| lithocholic acid | 0.7 | 0.0756 | lithocholic acid | 1.1 | 0.6199 | lithocholic acid | 0.6 | 0.1386 |
| **steroids** |  |  | **steroids** |  |  | **steroids** |  |  |
| corticosterone | 1.2 | 0.1974 | corticosterone | 1.2 | 0.1394 | corticosterone | 1.2 | 0.1417 |
| cortisol | 1.1 | 0.0854 | cortisol | 1.2 | 0.0521 | cortisol | 1.3 | **0.0051** |
| cortisone | 1.2 | **0.0054** | cortisone | 1 | 0.3228 | cortisone | 1 | 0.6957 |

| cohort C |  |  | cohort C |  |  | cohort C |  |  |
| --- | --- | --- | --- | --- | --- | --- | --- | --- |
| PD vs CN |  |  | PSP vs CN |  |  | MSA vs CN |  |  |
|  | ratio | p value |  | ratio | p value |  | ratio | p value |
| **PAF** | 0.9 | **0.0061** | **PAF** | 0.8 | 0.0036 | **PAF** | 0.8 | **0.0019** |
| **acylethanolamine** | 0.6 | 0.1959 | **acylethanolamine** | 0.6 | 0.1917 | **acylethanolamine** | 0.6 | 0.2118 |
| **thyroid hormone** |  |  | **thyroid hormone** |  |  | **thyroid hormone** |  |  |
| thyroxine | 1.3 | **0.0074** | thyroxine | 1.2 | **0.0135** | thyroxine | 1.2 | **0.0240** |
| triiodothyronine | 1.2 | 0.2475 | triiodothyronine | 1.1 | 0.4140 | triiodothyronine | 0.9 | 0.3577 |
| **cholic acids** |  |  | **cholic acids** |  |  | **cholic acids** |  |  |
| ursodeoxycholic acid | 0.4 | 0.0651 | ursodeoxycholic acid | 5.8 | 0.1491 | ursodeoxycholic acid | 0.4 | 0.0746 |
| chenodeoxycholic acid | 0.9 | 0.5749 | chenodeoxycholic acid | 1.6 | 0.2746 | chenodeoxycholic acid | 0.2 | 0.1051 |
| cholic acid | 0.6 | 0.3441 | cholic acid | 1.3 | 0.3896 | cholic acid | 0.2 | 0.2015 |
| deoxycholic acid | 0.8 | 0.6290 | deoxycholic acid | 1 | 0.5139 | deoxycholic acid | 0.5 | 0.1928 |
| lithocholic acid | 0.6 | 0.2232 | lithocholic acid | 0.5 | 0.1522 | lithocholic acid | 0.5 | 0.1422 |
| **steroids** |  |  | **steroids** |  |  | **steroids** |  |  |
| Corticosterone | 1 | 0.4586 | Corticosterone | 1.2 | 0.3038 | Corticosterone | 0.9 | 0.6421 |
| Cortisol | 1.2 | 0.1064 | Cortisol | 1.3 | **0.0143** | Cortisol | 1.2 | 0.1288 |
| Cortisone | 1.2 | **0.0295** | Cortisone | 1.2 | **0.0490** | Cortisone | 1.3 | **0.0295** |

Statistical methods: The metabolite level ratio of IPD, DLB, MSA, AD, or PSP to CNs. Statistical significance was examined using one-tailed Welch's t tests (P < 0.05).

Abbreviations: platelet-activating factor (PAF)

*(iii) The collection of probands remains on very low, which lowers the impact of the manuscript.
Editor:  Please respond to this comment.*

Thank you for your suggestion. We considered the collection of probands to be genetic analysis.

We could not test GBA, LRRK2, and SNCA mutations in this study, so we changed sentences in Discussion section. Changes in the revised manuscript are highlighted in yellow.

First, Analysis the major causative genes or risk genes of PD during lipidomics were not evaluated. GBA1 mutations were not evaluated in all enrolled IPD patients. Based on the GBA1 genotype and clinical analysis, it has been reported that GBA1 mutation is the most common genetic risk factor for IPD patients, accounting for as many as 7% of all IPD patients in multicenter analyses [1,2]. On the other hand, only approximately 3% of Asian IPD patients with no apparent family history of parkinsonism are GBA1 mutation carriers [27]. IPD in GBA1 mutation carriers generally has an early onset [28]. However, there was no apparent family history of parkinsonism or dementia in all enrolled IPD patients, with a later mean age of onset in the enrolled IPD patients that was 67.2 years in cohort A and 65.2 years in cohort C. Elevated plasma GlcCer levels have recently been reported in GBA1 mutation carriers of IPD. Elevated plasma GlcCer levels have also been reported in non-GBA1 mutation carriers of IPD. These indicate that elevated plasma GlcCer is found in IPD with or without GBA mutation. Taken together, it is not plausible that a GBA1 mutation did not significantly affect elevated plasma GlcCer (a MonCer) levels in the IPD patients in this study. In addition, in this study LRRK2 and SNCA mutations, the major causative genes of PD, were not evaluated in all enrolled IPD patients. Analysis the major causative genes or risk genes of PD during lipidomics need to be performed in future studies. (p31-32, lines 491-507)

In addition, we considered the collection of probands to be the number of cases, so we changed sentences in Discussion section.

Second, this study is a small cases and cross-sectional study that could not account for multiple comparisons for several analytes detected in plasma. Future additional cases and longitudinal studies need to be performed. (p32, lines 507-509)

*(iv) The authors should be careful in claiming that the SL are similar in plasma compared to CSF or brain as was questioned by a reviewer; the papers the authors refer to are indirect and one even identified different findings in serum versus CSF. Perhaps it is better to include this in the limitations that these findings need to be confirmed in CSF or brain for further validation.*

*Editor: please address this comment.*

Thank you for your suggestion. We changed sentence in Discussion section as you indicated. Changes in the revised manuscript are highlighted in yellow.

Eighth, the increase and decrease in CSF sphingolipids and blood sphingolipids have coincided [30,31,58,59] in previous reports. On the other hand, one report even identified different findings in serum versus CSF [29,30]. These reports are indirect and sphingolipids need to be confirmed in CSF or brain for further validation. (p33, lines 523-526)

*(v) In addition, a few other questions are still open. Did the authors check for different lipid species as different chain length is important to define their function and hence a more in-depth analyses provides a better understanding of the findings.*

*Editor: please address this question.*

Thank you for your suggestion. To check for different lipid species as different chain length, we examined the association between lipid abnormalities and chain length in MonCers and LacCers.

We added sentences in Results section and additional Table (Table 2 and Table 3). Changes in the revised manuscript are highlighted in yellow.

We compared MonCer (d18:1/14:0), MonCer (d18:1/16:0), MonCer (d18:1/16:1), MonCer (d18:1/18:0), MonCer (d18:1/18:1), MonCer (d18:1/20:0), MonCer (d18:1/20:1), MonCer (d18:1/22:0), MonCer (d18:1/22:1), MonCer (d18:1/22:2), MonCer (d18:1/24:0), MonCer (d18:1/24:1), and MonCer (d18:1/24:2) between the CN group and the IPD, DLB, MSA, AD, or PSP groups (Table 2). The χ-square test was used to examine the association between lipid abnormalities and chain length in MonCer d18:1. No statistically significant difference was found between lipid abnormalities and chain length (P = 0.5522) in all enrolled groups. (p16, lines 261-268)

We compared LacCer (d18:1/14:0), LacCer (d18:1/16:0), LacCer (d18:1/16:1), LacCer (d18:1/18:0), LacCer (d18:1/18:1), LacCer (d18:1/20:0), LacCer (d18:1/20:1), LacCer (d18:1/22:0), LacCer (d18:1/22:1), LacCer (d18:1/22:2), LacCer (d18:1/24:0), LacCer (d18:1/24:1), and LacCer (d18:1/24:2) between the CN group and the IPD, DLB, MSA, AD, or PSP groups (Table 3). The χ-square test was used to examine the association between lipid abnormalities and chain length in LacCers d18:1. No statistically significant difference was found between lipid abnormalities and chain length (P = 0.5522) in all enrolled groups. (p17-18, lines 296-302)

**Table 2. Plasma All MonCer Levels in Neurodegenerative Diseases.**

| cohort A |  |  | cohort B |  |  | cohort B |  |  |
| --- | --- | --- | --- | --- | --- | --- | --- | --- |
| PD vs CN |  |  | DLB vs CN |  |  | AD vs CN |  |  |
|  | ratio | p value |  | ratio | p value |  | ratio | p value |
| MonCer (d18:1/14:0) | 1.1 | 0.0975 | MonCer (d18:1/14:0) | 1.3 | **0.0012** | MonCer (d18:1/14:0) | 1.2 | **0.0443** |
| MonCer (d18:1/16:0) | 1.2 | **0.0151** | MonCer (d18:1/16:0) | 1.2 | **0.0013** | MonCer (d18:1/16:0) | 1.3 | **0.0023** |
| MonCer (d18:1/16:1) | 1 | 0.5408 | MonCer (d18:1/16:1) | 1.1 | 0.2052 | MonCer (d18:1/16:1) | 1 | 0.4006 |
| MonCer (d18:1/18:0) | 1.3 | **0.0105** | MonCer (d18:1/18:0) | 1.2 | **0.0181** | MonCer (d18:1/18:0) | 1.3 | **0.0008** |
| MonCer (d18:1/18:1) | 1.4 | **0.0018** | MonCer (d18:1/18:1) | 1.3 | **0.0042** | MonCer (d18:1/18:1) | 1.3 | **0.0106** |
| MonCer (d18:1/20:0) | 1.2 | **0.0053** | MonCer (d18:1/20:0) | 1.1 | **0.0890** | MonCer (d18:1/20:0) | 1.2 | **0.0298** |
| MonCer (d18:1/20:1) | 1.5 | **0.0010** | MonCer (d18:1/20:1) | 1.4 | **0.0066** | MonCer (d18:1/20:1) | 1.4 | **0.0047** |
| MonCer (d18:1/22:0) | 1.1 | 0.2312 | MonCer (d18:1/22:0) | 1 | 0.5454 | MonCer (d18:1/22:0) | 1.1 | 0.2288 |
| MonCer (d18:1/22:1) | 1.4 | **0.0092** | MonCer (d18:1/22:1) | 1.5 | **0.0025** | MonCer (d18:1/22:1) | 1.5 | **0.0121** |
| MonCer (d18:1/22:2) | 1.3 | **0.0097** | MonCer (d18:1/22:2) | 1.4 | **0.0034** | MonCer (d18:1/22:2) | 1.5 | **0.0039** |
| MonCer (d18:1/24:0) | 1.1 | 0.2478 | MonCer (d18:1/24:0) | 1 | 0.5483 | MonCer (d18:1/24:0) | 0.9 | 0.6499 |
| MonCer (d18:1/24:1) | 1.3 | **0.0016** | MonCer (d18:1/24:1) | 1.3 | **0.0143** | MonCer (d18:1/24:1) | 1.3 | **0.0244** |
| MonCer (d18:1/24:2) | 1.2 | **0.0176** | MonCer (d18:1/24:2) | 1.2 | **0.0170** | MonCer (d18:1/24:2) | 1.4 | **0.0313** |

| cohort C |  |  | cohort C |  |  | cohort C |  |  |
| --- | --- | --- | --- | --- | --- | --- | --- | --- |
| PD vs CN |  |  | PSP vs CN |  |  | MSA vs CN |  |  |
|  | ratio | p value |  | ratio | p value |  | ratio | p value |
| MonCer (d18:1/14:0) | 1.3 | 0.0826 | MonCer (d18:1/14:0) | 1.3 | 0.0709 | MonCer (d18:1/14:0) | 1.2 | 0.1345 |
| MonCer (d18:1/16:0) | 1.4 | **0.0029** | MonCer (d18:1/16:0) | 1.4 | **0.0035** | MonCer (d18:1/16:0) | 1.4 | **0.0099** |
| MonCer (d18:1/16:1) | 1.2 | 0.3156 | MonCer (d18:1/16:1) | 1.5 | 0.1082 | MonCer (d18:1/16:1) | 1.3 | 0.2442 |
| MonCer (d18:1/18:0) | 1.5 | **0.0018** | MonCer (d18:1/18:0) | 1.5 | **0.0030** | MonCer (d18:1/18:0) | 1.4 | **0.0074** |
| MonCer (d18:1/18:1) | 1.3 | **0.0018** | MonCer (d18:1/18:1) | 1.5 | **0.0006** | MonCer (d18:1/18:1) | 1.2 | **0.0910** |
| MonCer (d18:1/20:0) | 1.3 | **0.0267** | MonCer (d18:1/20:0) | 1.4 | **0.0100** | MonCer (d18:1/20:0) | 1.3 | **0.0430** |
| MonCer (d18:1/20:1) | 1.3 | **0.0182** | MonCer (d18:1/20:1) | 1.5 | **0.0020** | MonCer (d18:1/20:1) | 1.3 | **0.0172** |
| MonCer (d18:1/22:0) | 0.8 | 0.7890 | MonCer (d18:1/22:0) | 1.1 | 0.1771 | MonCer (d18:1/22:0) | 0.9 | 0.7486 |
| MonCer (d18:1/22:1) | 1 | 0.5938 | MonCer (d18:1/22:1) | 1.4 | 0.0729 | MonCer (d18:1/22:1) | 1.2 | 0.2142 |
| MonCer (d18:1/22:2) | 1.5 | **0.0009** | MonCer (d18:1/22:2) | 1.6 | **0.0013** | MonCer (d18:1/22:2) | 1.6 | **0.0189** |
| MonCer (d18:1/24:0) | 0.4 | **<0.0001** | MonCer (d18:1/24:0) | 1.1 | 0.2573 | MonCer (d18:1/24:0) | 0.7 | **0.0158** |
| MonCer (d18:1/24:1) | 0.9 | 0.2863 | MonCer (d18:1/24:1) | 1.4 | **0.0136** | MonCer (d18:1/24:1) | 1.1 | 0.3389 |
| MonCer (d18:1/24:2) | 1.2 | 0.0725 | MonCer (d18:1/24:2) | 1.5 | **0.0145** | MonCer (d18:1/24:2) | 1.3 | 0.0697 |

Statistical methods: The metabolite level ratio of IPD, DLB, MSA, AD, or PSP to CNs. Statistical significance was examined using one-tailed Welch's t tests (P < 0.05). (p21-22, lines 347-352)

**Table 3. Plasma All LacCer Levels in Neurodegenerative Diseases.**

| cohort A |  |  | cohort B |  |  | cohort B |  |  |
| --- | --- | --- | --- | --- | --- | --- | --- | --- |
| PD vs CN |  |  | DLB vs CN |  |  | AD vs CN |  |  |
|  | ratio | p value |  | ratio | p value |  | ratio | p value |
| LacCer (d18:1/14:0) | 1.2 | **0.0035** | LacCer (d18:1/14:0) | 1.5 | **<0.0001** | LacCer (d18:1/14:0) | 1.2 | **0.0297** |
| LacCer (d18:1/16:0) | 1.2 | **0.0026** | LacCer (d18:1/16:0) | 1.2 | **0.0006** | LacCer (d18:1/16:0) | 1.2 | **0.0284** |
| LacCer (d18:1/16:1) | 1.2 | **0.0161** | LacCer (d18:1/16:1) | 1.3 | **0.0002** | LacCer (d18:1/16:1) | 1.2 | 0.0655 |
| LacCer (d18:1/18:0) | 1.1 | 0.1188 | LacCer (d18:1/18:0) | 1.2 | **0.0110** | LacCer (d18:1/18:0) | 1.2 | **0.0340** |
| LacCer (d18:1/18:1) | 1.3 | **0.0024** | LacCer (d18:1/18:1) | 1.2 | **0.0278** | LacCer (d18:1/18:1) | 1.2 | 0.0850 |
| LacCer (d18:1/20:0) | 1 | 0.3364 | LacCer (d18:1/20:0) | 1.1 | 0.1147 | LacCer (d18:1/20:0) | 1.2 | 0.1116 |
| LacCer (d18:1/20:1) | 1.4 | **0.0032** | LacCer (d18:1/20:1) | 1.4 | **0.0009** | LacCer (d18:1/20:1) | 1.3 | **0.0179** |
| LacCer (d18:1/22:0) | 1 | 0.5292 | LacCer (d18:1/22:0) | 1.1 | 0.2660 | LacCer (d18:1/22:0) | 1.1 | 0.2379 |
| LacCer (d18:1/22:1) | 1.3 | **0.0095** | LacCer (d18:1/22:1) | 1.5 | **<0.0001** | LacCer (d18:1/22:1) | 1.4 | **0.0167** |
| LacCer (d18:1/22:2) | 1.3 | **0.0018** | LacCer (d18:1/22:2) | 1.3 | **0.0062** | LacCer (d18:1/22:2) | 1.3 | **0.0268** |
| LacCer (d18:1/24:0) | 1 | 0.4800 | LacCer (d18:1/24:0) | 1.1 | 0.3120 | LacCer (d18:1/24:0) | 1 | 0.6157 |
| LacCer (d18:1/24:1) | 1.4 | **0.0003** | LacCer (d18:1/24:1) | 1.3 | **0.0082** | LacCer (d18:1/24:1) | 1.2 | 0.1190 |
| LacCer (d18:1/24:2) | 1.3 | **0.0021** | LacCer (d18:1/24:2) | 1.3 | **0.0098** | LacCer (d18:1/24:2) | 1.1 | 0.1727 |

| cohort C |  |  | cohort C |  |  | cohort C |  |  |
| --- | --- | --- | --- | --- | --- | --- | --- | --- |
| PD vs CN |  |  | PSP vs CN |  |  | MSA vs CN |  |  |
|  | ratio | p value |  | ratio | p value |  | ratio | p value |
| LacCer (d18:1/14:0) | 1.4 | **0.0252** | LacCer (d18:1/14:0) | 1.3 | **0.0320** | LacCer (d18:1/14:0) | 1.4 | **0.0194** |
| LacCer (d18:1/16:0) | 1.3 | **0.0063** | LacCer (d18:1/16:0) | 1.4 | **0.0019** | LacCer (d18:1/16:0) | 1.3 | **0.0059** |
| LacCer (d18:1/16:1) | 1.2 | **0.0157** | LacCer (d18:1/16:1) | 1.5 | **0.0178** | LacCer (d18:1/16:1) | 1.2 | **0.0454** |
| LacCer (d18:1/18:0) | 1.2 | 0.0953 | LacCer (d18:1/18:0) | 1.5 | 0.2933 | LacCer (d18:1/18:0) | 1.3 | **0.0392** |
| LacCer (d18:1/18:1) | 1.2 | 0.1763 | LacCer (d18:1/18:1) | 1.5 | 0.1542 | LacCer (d18:1/18:1) | 1 | 0.4351 |
| LacCer (d18:1/20:0) | 1.1 | 0.2281 | LacCer (d18:1/20:0) | 1.4 | 0.3886 | LacCer (d18:1/20:0) | 1.2 | 0.1381 |
| LacCer (d18:1/20:1) | 1.2 | 0.0902 | LacCer (d18:1/20:1) | 1.5 | 0.2285 | LacCer (d18:1/20:1) | 1.2 | 0.1321 |
| LacCer (d18:1/22:0) | 0.8 | 0.1630 | LacCer (d18:1/22:0) | 1.1 | 0.6517 | LacCer (d18:1/22:0) | 0.9 | 0.2980 |
| LacCer (d18:1/22:1) | 1.1 | 0.3525 | LacCer (d18:1/22:1) | 1.4 | 0.2833 | LacCer (d18:1/22:1) | 1.1 | 0.2590 |
| LacCer (d18:1/22:2) | 1.3 | 0.0925 | LacCer (d18:1/22:2) | 1.6 | 0.1774 | LacCer (d18:1/22:2) | 1.2 | 0.1781 |
| LacCer (d18:1/24:0) | 0.4 | **0.0031** | LacCer (d18:1/24:0) | 1.1 | 0.7710 | LacCer (d18:1/24:0) | 0.7 | **0.0248** |
| LacCer (d18:1/24:1) | 0.9 | 0.6587 | LacCer (d18:1/24:1) | 1.4 | 0.3145 | LacCer (d18:1/24:1) | 1 | 0.4450 |
| LacCer (d18:1/24:2) | 1.2 | 0.2138 | LacCer (d18:1/24:2) | 1.5 | 0.2520 | LacCer (d18:1/24:2) | 1.2 | 0.2394 |

Statistical methods: The metabolite level ratio of IPD, DLB, MSA, AD, or PSP to CNs. Statistical significance was examined using one-tailed Welch's t tests (P < 0.05). (p23-24, lines 354-359)

*(vi) Can the authors test if the IPD samples contain mutations in LRRK2 and SNCA as these are regularly identified in IPD and may provide a link to genetic forms of PD*

*Editor:* *Can you test for these mutations; doing so would definitely increase the impact of your paper.*

Thank you for your suggestion. However, we could not test LRRK2, and SNCA mutations in this study.

We added sentences in Discussion section. Changes in the revised manuscript are highlighted in yellow.

In addition, in this study LRRK2 and SNCA mutations, the major causative genes of PD, were not evaluated in all enrolled IPD patients. Analysis the major causative genes or risk genes of PD during lipidomics need to be performed in future studies. (p32, lines 504-507)

*5) Editor: lines 427-431, revised: "Seventh, Quantitative data were not used as metabolites values in this study. In present lipidomic analysis, the coefficient of variation (CV) ranged from 4.4 to 9.7%, with a mean of 6.7%. Because of the variability of metabolites values, present method should be compared with healthy subjects for each cohort. Therefore, the relative areas were used as metabolites values in this study."*

*Editor: The above explanation is very hard to understand.*

Thank you for your suggestion. We agree with your opinion. We measured relative areas as quantitative value based on PROS ONE manuscript [10]. Lipidomics has the variability of metabolite values in each study. For this reason, each metabolite should be normalized based on the internal standard (IS) level and sample volume. The normalized each metabolite was represented as relative area and used as the quantitative value.

We changed sentence in Discussion section as you indicated. Changes in the revised manuscript are highlighted in yellow.

Seventh, relative area was used in this study as the quantitative value for each metabolite based on previous reports [10,14]. Lipidomics has the variability of metabolite values in each study. For this reason, each metabolite should be normalized based on the IS level and sample volume. The normalized each metabolite was represented as relative area and used as the quantitative value. (p32-33, lines 518-522)

1.	Sidransky E, Nalls MA, Aasly JO, Aharon-Peretz J, Annesi G, Barbosa ER, et al. Multicenter analysis of glucocerebrosidase mutations in Parkinson's disease. N Engl J Med. 2009;361: 1651-1661.

2.	Lesage S, Anheim M, Condroyer C, Pollak P, Durif F, Dupuits C, et al. Large-scale screening of the Gaucher's disease-related glucocerebrosidase gene in Europeans with Parkinson's disease. Hum Mol Genet. 2011;20: 202-210.

3.	Foo JN, Liany H, Bei JX, Yu XQ, Liu J, Au WL, et al. Rare lysosomal enzyme gene SMPD1 variant (p.R591C) associates with Parkinson's disease. Neurobiol Aging. 2013;34: 2890 e2813-2895.

4.	Kluenemann HH, Nutt JG, Davis MY, Bird TD. Parkinsonism syndrome in heterozygotes for Niemann-Pick C1. J Neurol Sci. 2013;335: 219-220.

5.	Gregory A, Westaway SK, Holm IE, Kotzbauer PT, Hogarth P, Sonek S, et al. Neurodegeneration associated with genetic defects in phospholipase A(2). Neurology. 2008;71: 1402-1409.

6.	Chartier-Harlin MC, Parfitt M, Legrain S, Pérez-Tur J, Brousseau T, Evans A, et al. Apolipoprotein E, epsilon 4 allele as a major risk factor for sporadic early and late-onset forms of Alzheimer's disease: analysis of the 19q13.2 chromosomal region. Hum Mol Genet. 1994;3: 569-574.

7.	Yamazaki T, Chang TY, Haass C, Ihara Y. Accumulation and aggregation of amyloid beta-protein in late endosomes of Niemann-pick type C cells. J Biol Chem. 2001;276: 4454-4460.

8.	Yin F. Lipid metabolism and Alzheimer's disease: clinical evidence, mechanistic link and therapeutic promise. FEBS J. 2022.

9.	Xicoy H, Wieringa B, Martens GJM. The Role of Lipids in Parkinson's Disease. Cells. 2019;8.

10.	Mori A, Ishikawa KI, Saiki S, Hatano T, Oji Y, Okuzumi A, et al. Plasma metabolite biomarkers for multiple system atrophy and progressive supranuclear palsy. PLoS One. 2019;14: e0223113.

11.	Hayasaka R, Tabata S, Hasebe M, Ikeda S, Ohnuma S, Mori M, et al. Metabolomic Analysis of Small Extracellular Vesicles Derived from Pancreatic Cancer Cells Cultured under Normoxia and Hypoxia. Metabolites. 2021;11.

12.	Suzuki Y, Hayasaka R, Hasebe M, Ikeda S, Soga T, Tomita M, et al. Comparative Metabolomics of Small Molecules Specifically Expressed in the Dorsal or Ventral Marginal Zones in Vertebrate Gastrula. Metabolites. 2022;12.

13.	Ikeda K. Mass Spectrometric Analysis of Phospholipids by Target Discovery Approach. 2015. pp. 349-356.

14.	Saiki S, Sasazawa Y, Fujimaki M, Kamagata K, Kaga N, Taka H, et al. A metabolic profile of polyamines in parkinson disease: A promising biomarker. Ann Neurol. 2019;86: 251-263.

15.	Merrill AH, Jr. Sphingolipid and glycosphingolipid metabolic pathways in the era of sphingolipidomics. Chem Rev. 2011;111: 6387-6422.

16.	Yamaji T, Hanada K. Sphingolipid metabolism and interorganellar transport: localization of sphingolipid enzymes and lipid transfer proteins. Traffic. 2015;16: 101-122.

17.	Chan RB, Perotte AJ, Zhou B, Liong C, Shorr EJ, Marder KS, et al. Elevated GM3 plasma concentration in idiopathic Parkinson's disease: A lipidomic analysis. PLoS One. 2017;12: e0172348.

18.	Sohn H, Kim YS, Kim HT, Kim CH, Cho EW, Kang HY, et al. Ganglioside GM3 is involved in neuronal cell death. FASEB J. 2006;20: 1248-1250.

19.	De Maria R, Lenti L, Malisan F, d'Agostino F, Tomassini B, Zeuner A, et al. Requirement for GD3 ganglioside in CD95- and ceramide-induced apoptosis. Science. 1997;277: 1652-1655.

20.	Schengrund CL. Lipid rafts: keys to neurodegeneration. Brain Res Bull. 2010;82: 7-17.

21.	Wu G, Lu ZH, Kulkarni N, Amin R, Ledeen RW. Mice lacking major brain gangliosides develop parkinsonism. Neurochem Res. 2011;36: 1706-1714.

22.	Chalfant CE, Spiegel S. Sphingosine 1-phosphate and ceramide 1-phosphate: expanding roles in cell signaling. J Cell Sci. 2005;118: 4605-4612.

23.	Arana L, Gangoiti P, Ouro A, Trueba M, Gomez-Munoz A. Ceramide and ceramide 1-phosphate in health and disease. Lipids Health Dis. 2010;9: 15.

24.	Leng F, Edison P. Neuroinflammation and microglial activation in Alzheimer disease: where do we go from here? Nat Rev Neurol. 2021;17: 157-172.

25.	Gelders G, Baekelandt V, Van der Perren A. Linking Neuroinflammation and Neurodegeneration in Parkinson's Disease. J Immunol Res. 2018;2018: 4784268.

26.	Surendranathan A, Rowe JB, O'Brien JT. Neuroinflammation in Lewy body dementia. Parkinsonism Relat Disord. 2015;21: 1398-1406.

27.	Wu YR, Chen CM, Chao CY, Ro LS, Lyu RK, Chang KH, et al. Glucocerebrosidase gene mutation is a risk factor for early onset of Parkinson disease among Taiwanese. J Neurol Neurosurg Psychiatry. 2007;78: 977-979.

28.	Sato C, Morgan A, Lang AE, Salehi-Rad S, Kawarai T, Meng Y, et al. Analysis of the glucocerebrosidase gene in Parkinson's disease. Mov Disord. 2005;20: 367-370.

29.	Mielke MM, Maetzler W, Haughey NJ, Bandaru VV, Savica R, Deuschle C, et al. Plasma ceramide and glucosylceramide metabolism is altered in sporadic Parkinson's disease and associated with cognitive impairment: a pilot study. PLoS One. 2013;8: e73094.

30.	Huh YE, Park H, Chiang MSR, Tuncali I, Liu G, Locascio JJ, et al. Glucosylceramide in cerebrospinal fluid of patients with GBA-associated and idiopathic Parkinson's disease enrolled in PPMI. NPJ Parkinsons Dis. 2021;7: 102.
