# Supplementary material for: Plasma sphingolipid abnormalities in neurodegenerative diseases
Source: PLoS One. 2022 Dec 16;17(12):e0279315. doi: 10.1371/journal.pone.0279315 (PMC9757566; doi:10.1371/journal.pone.0279315)
Supplement: Attachment — Submitted filename: Response to reviewers20221202.docx [file pone.0279315.s006.docx]

*Additional Editor Comments :*

*I will accept your manuscript after you make corrections to S1 Table. In S1 Table, you repeatedly misspell the word "sphingolipids." Throughout the table sphingolipids is spelled "sphinogolipids." Please fix.*

Thank you for your suggestion. We have changed the misspelled "sphinogolipids." in S1 Table and Supporting information to correct "sphingolipids." Changes in the revised manuscript are highlighted in yellow.

S1 Table. Plasma other sphingolipids, sphinganines, gangliosides, free fatty acids, acylcarnitnes, lysophospholipids levels in neurodegenerative diseases. (p47, lines 758-759)

**S1 Table. Plasma Other sphingolipids, Sphinganines, Gangliosides, Free fatty acids, Acylcarnitnes, Lysophospholipids Levels in Neurodegenerative Diseases.**

| cohort A |  |  | cohort B |  |  | cohort B |  |  |
| --- | --- | --- | --- | --- | --- | --- | --- | --- |
| PD vs CN |  |  | DLB vs CN |  |  | AD vs CN |  |  |
|  | ratio | p value |  | ratio | p value |  | ratio | p value |
| **other sphingolipids** |  |  | **other sphingolipids** |  |  | **other sphingolipids** |  |  |
| C1P | 1.1 | **0.0291** | C1P | 1.2 | **0.0004** | C1P | 1.3 | **0.0001** |
| sphingosine | 0.8 | **0.0436** | sphingosine | 0.7 | 0.0586 | sphingosine | 0.8 | 0.1119 |
| **sphinganines** |  |  | **sphinganines** |  |  | **sphinganines** |  |  |
| sphinganine | 0.8 | **0.0031** | sphinganine | 0.8 | **0.0191** | sphinganine | 0.8 | **0.0248** |
| SG1P | 0.9 | 0.0535 | SG1P | 0.8 | **0.0312** | SG1P | 0.8 | **0.0284** |
| **gangliosides** |  |  | **gangliosides** |  |  | **gangliosides** |  |  |
| GM3 | 1.1 | **0.0169** | GM3 | 1.2 | **0.0001** | GM3 | 1.3 | **<0.0001** |
| GD3 | 1.1 | **0.0483** | GD3 | 1.3 | **<0.0001** | GD3 | 1.4 | **<0.0001** |
| GD1 | 0.9 | 0.1471 | GD1 | 0.9 | 0.0927 | GD1 | 1.1 | 0.3198 |
| **free fatty acids** | 1.6 | **0.0174** | **free fatty acids** | 1.5 | 0.0502 | **free fatty acids** | 1.2 | 0.2940 |
| **acylcarnitnes** | 1.2 | 0.1048 | **acylcarnitnes** | 1.6 | **0.0014** | **acylcarnitnes** | 1.4 | 0.0580 |
| **lysophospholipids** |  |  | **lysophospholipids** |  |  | **lysophospholipids** |  |  |
| LPA | 0.9 | **0.0458** | LPA | 0.7 | **0.0092** | LPA | 0.9 | 0.3597 |
| LPC | 1 | 0.5986 | LPC | 0.8 | **0.0347** | LPC | 0.9 | 0.1295 |
| LPE | 1 | 0.5126 | LPE | 0.7 | **0.0034** | LPE | 0.9 | 0.2807 |
| LPG | 0.9 | **0.0032** | LPG | 0.8 | **0.0019** | LPG | 0.9 | 0.0453 |
| LPI | 0.9 | **0.0218** | LPI | 0.9 | 0.0749 | LPI | 1.1 | 0.3255 |
| LPS | 0.8 | **0.0366** | LPS | 0.6 | **0.0335** | LPS | 0.6 | 0.0734 |

| cohort C |  |  | cohort C |  |  | cohort C |  |  |
| --- | --- | --- | --- | --- | --- | --- | --- | --- |
| PD vs CN |  |  | PSP vs CN |  |  | MSA vs CN |  |  |
|  | ratio | p value |  | ratio | p value |  | ratio | p value |
| **other sphingolipids** |  |  | **other sphingolipids** |  |  | **other sphingolipids** |  |  |
| C1P | 1.3 | **0.0245** | C1P | 1.1 | 0.1851 | C1P | 1.2 | 0.1301 |
| sphingosine | 0.9 | 0.2337 | sphingosine | 0.7 | 0.0669 | sphingosine | 0.8 | 0.0902 |
| **sphinganines** |  |  | **sphinganines** |  |  | **sphinganines** |  |  |
| sphinganine | 0.9 | 0.8344 | sphinganine | 0.8 | 0.0718 | sphinganine | 0.9 | 0.1685 |
| SG1P | 0.7 | 0.0615 | SG1P | 0.7 | 0.0372 | SG1P | 0.7 | 0.0635 |
| **gangliosides** |  |  | **gangliosides** |  |  | **gangliosides** |  |  |
| GM3 | 1.3 | **<0.0001** | GM3 | 1.2 | **0.0005** | GM3 | 1.2 | **0.0012** |
| GD3 | 1.5 | **<0.0001** | GD3 | 1.2 | **0.0136** | GD3 | 1.1 | **<0.0001** |
| GD1 | 0.9 | 0.7283 | GD1 | 1.1 | 0.3171 | GD1 | 1.4 | 0.3579 |
| **free fatty acids** | 1.5 | 0.1049 | **free fatty acids** | 1.4 | 0.2088 | **free fatty acids** | 1.5 | 0.1559 |
| **acylcarnitnes** | 1.1 | 0.2606 | **acylcarnitnes** | 1.2 | 0.2046 | **acylcarnitnes** | 1.3 | 0.1451 |
| **lysophospholipids** |  |  | **lysophospholipids** |  |  | **lysophospholipids** |  |  |
| LPA | 1 | 0.5317 | LPA | 0.7 | **0.0308** | LPA | 1 | 0.5593 |
| LPC | 0.9 | **0.0335** | LPC | 0.9 | **0.0427** | LPC | 0.8 | **0.0128** |
| LPE | 0.8 | 0.0884 | LPE | 0.8 | 0.0532 | LPE | 0.7 | 0.0113 |
| LPG | 0.8 | 0.0645 | LPG | 0.8 | 0.0461 | LPG | 0.8 | **0.0423** |
| LPI | 1.1 | 0.1846 | LPI | 1 | 0.5590 | LPI | 1 | 0.5501 |
| LPS | 0.9 | 0.3106 | LPS | 0.7 | 0.1019 | LPS | 0.7 | 0.1078 |

Statistical methods: The metabolite level ratio of IPD, DLB, MSA, AD, or PSP to CNs. Statistical significance was examined using one-tailed Welch's t tests (P < 0.05).

Abbreviations: ceramide-1-phosphate (C1P), sphinganine-1-phosphate (SG1P), lysophosphatidic acid (LPA), lysophosphatidylcholine (LPC), lysophosphatidylethanolamine (LPE), lysophosphatidylglycerol (LPG), lysophosphatidylinositol (LPI), lysophosphatidylserine (LPS)
